# Evaluation of the Risk Factors for Cellulitis among Patients with Peripheral Artery Disease

**DOI:** 10.3390/medicina59050933

**Published:** 2023-05-12

**Authors:** Bo-Yuan Wang, Shun-Fa Yang, Ke-Hsin Ting, Yu-Hsun Wang, Ming-Chih Chou, Chao-Bin Yeh

**Affiliations:** 1 Institute of Medicine, Chung Shan Medical University, Taichung 402, Taiwan; 2 School of Medicine, Chung Shan Medical University, Taichung 402, Taiwan; 3 Department of Emergency Medicine, Chung Shan Medical University Hospital, Taichung 402, Taiwan; 4 Department of Medical Research, Chung Shan Medical University Hospital, Taichung 402, Taiwan; cshe731@csh.org.tw; 5 Division of Cardiology, Department of Internal Medicine, Changhua Christian Hospital, Yunlin Branch, Changhua 648, Taiwan; 6 Department of Post-Baccalaureate Medicine, College of Medicine, National Chung Hsing University, Taichung 402, Taiwan; 7 Department of Surgery, Chung Shan Medical University Hospital, Taichung 402, Taiwan

**Keywords:** peripheral occlusion artery disease (PAOD), cellulitis, population-based cohort study

## Abstract

*Background and objectives*: The objective of this study is to elucidate peripheral occlusion artery disease (PAOD) as a risk factor for cellulitis. *Materials and Methods*: This is a retrospective population-based cohort study. The database is the Longitudinal Health Insurance Database, which covers two million beneficiaries from the entire population of the 2010 registry for beneficiaries in Taiwan. The PAOD group is composed of patients who were newly diagnosed with PAOD from 2001 to 2014. The non-PAOD group is composed of patients who were never diagnosed with PAOD from 2001 to 2015. All patients were followed until the onset of cellulitis, death, or until the end of 2015. *Results*: Finally, 29,830 patients who were newly diagnosed with PAOD were included in the PAOD group, and 29,830 patients who were never diagnosed with PAOD were included in the non-PAOD group. The incidence densities (ID) of cellulitis were 26.05 (95% CI = 25.31–26.80) patients per 1000 person-years in the PAOD group and 49.10 (95% CI = 48.04–50.19) in the non-PAOD group. The PAOD group had an increased risk of cellulitis (adjusted HR = 1.94, 95% CI = 1.87–2.01) compared to the non-PAOD group. *Conclusions*: Patients with PAOD were associated with a higher risk of subsequent cellulitis compared to patients without PAOD.

## 1. Introduction

Peripheral artery occlusion disease (PAOD), also known as peripheral artery disease (PAD), is a manifestation of atherosclerosis that causes the narrowing or occlusion of the arterial lumen. Blood flow and perfusion are impaired in the area of the distal part of the affected arteries [1]. Figures on the prevalence of PAOD vary with diagnostic criteria and the study population. The prevalence of PAOD was about 4.67–14% and up to 24.7% in individuals over the age of 70 [2,3,4]. It is estimated that over 200 million people have PAOD globally [5]. Cigarette smoking, diabetes, hypertension, hyperlipidemia, and age are the presently known risk factors [6,7,8]. Intermittent claudication (IC) is the most common symptom of PAOD, which manifests as leg pain that is induced by walking and relieved by rest. The severity of symptoms is determined by the extent of arterial stenosis, the number of affected arteries, and the intensity of exercise. Symptoms of PAOD in the male population were reported to be more severe than in the female population [6]. The diagnosis of PAD was also presented in the previous study [1].

Cellulitis is an infection of the subcutaneous tissue and deep dermis when indigenous flora enters the dermis and colonises the dermis by breaking the skin barrier; it manifests as an area of skin erythema, swelling, sensation of heat, and tenderness in the affected area [9]. The incidence of cellulitis varies by country. In the USA, approximately 25/1000 person-years was reported in 2006 [10]. In the Netherlands, the annual incidence was estimated to be 22/1000 person-years [11]. A study estimated that the incidence of cellulitis in the UK was 16.4/1000 person-years [12]. Cellulitis became a health burden, causing more than 650,000 hospitalizations and 3.7 billion USD in ambulatory care costs annually in the USA. Infectious organisms are identified in about 15% of cases; most of them are β-hemolytic Streptococcus and Staphylococcus aureus [9]. The infection can occur anywhere on the body, and the lower extremities are affected in most cases [13]. Cellulitis is usually thought to be caused by β-hemolytic Streptococcus and Staphylococcus aureus, and the presence of β-hemolytic Streptococcus and/or Staphylococcus aureus in the toe webs was strongly considered as a risk factor for cellulitis. Other risk factors associated with cellulitis include trauma, diabetes, smoking, chronic venous insufficiency, edema, destroyed skin barrier, previous history of bacterial cellulitis, existence of erosions or ulcers of the lower extremities, and a history of saphenectomy [13,14,15].

Although it remains controversial, there is increasing evidence linking skin lesions to infectious diseases [16,17,18]. A previous review article showed that patients with eosinophilic skin diseases, in which tissue layers or skin structures were affected by eosinophil infiltration and/or degranulation, had limited defense against certain parasitic infections [19,20]. Another meta-analysis showed that the pooled adjusted odds ratio of non-purulent cellulitis was more than 13 times higher in patients with current leg ulcers and 6 times higher in patients with excoriating skin diseases compared to patients in the control group [21]. In addition, maintaining the heath of skin tissue and protecting against microbial invasion depend on whether the functions of the blood vessels are normal or not. Impaired function of the skin microcirculation leads to being unable to maintain skin defense mechanisms. A comparative study described the relationship among skin lesions, vascular disease, and infection, and found that patients with chronic skin lesions and with peripheral vascular lesions simultaneously had differences in cutaneous bacterial flora compared to patients only with chronic skin lesions [17]. PAOD can cause arterial dysfunction and impaired blood perfusion; a progressive reduction of nutrition and oxygen supplement may change the physiological integrity of the skin tissue. According to the PAOD classification system, ischemia, tissue loss, ulcers, and even necrosis can occur as the disease progresses [22]. Therefore, PAOD patients are more susceptible to develop consequent cellulitis is a reasonable assumption, but research on the association between PAOD and cellulitis is still limited with only a few studies indicating this possibility without examining the time sequence between PAOD and cellulitis. Consequently, this study aimed to investigate the association of PAOD with the development of subsequent cellulitis.

## 2. Materials and Methods

### 2.1. Data Source

This retrospective, population-based cohort study was conducted using the dataset from the Longitudinal Health Insurance Database (LHID) 2010. LHID 2010 is an electronic database built based on the National Health Program that contains more than 99% of the population and is administered and regulated by the Health and Welfare Data Science Center in Taiwan. The database is composed of two million Taiwanese residents who were randomly enrolled and sampled by stratification according to age, sex, and region from the nearly entire Taiwan population of the 2010 registry in the mandatory National Health Insurance program. Its contents are comprised of patients’ information, such as the registry for beneficiaries, administrative claims in ambulatory care visits and admissions, diagnosis, medication, and interventions. All insurance claims were monitored by reimbursement specialists and peer reviewers, which made the diagnosis coding highly reliable. We can trace the medical record of patients from 2000 to 2015 by an analysis of the LHID 2010. This study protocol was reviewed by the Chung Shan Medical University Hospital ethics review board (CS1-20056).

### 2.2. Patient Selection and Outcome

The study sample was divided into two stratified cohorts based on the presence or absence of PAOD disease. Patients had newly diagnosed PAOD if they had received PAOD-related diagnostic codes (ICD-9-CM codes: 443.8, 443.9, and 444) in more than two outpatient visits or one admission, according to their medical records from 2001 to 2014, and comprised the PAOD group. The index date was the date of the first diagnosis of PAOD. To accurately explicate the association between PAOD and cellulitis, patients with a diagnosis of cellulitis before the index date were excluded. Patients without a PAOD diagnosis from 2000 to 2015 comprised the non-PAOD group. The outcome variable was the diagnosis of cellulitis, according to the diagnostic codes (ICD-9-CM codes: 376.01, 478.21, 478.71, 528.3, 614.3, 614.4, 681.0, 681, and 682). Both groups were followed-up until the onset of cellulitis, death, or 31 December 2015, whichever happened first.

### 2.3. Covariates and Matching

The demographic characteristics included in the analysis were age and sex and the comorbidities of hypertension, hyperlipidemia, diabetes, hyperthyroidism, hypothyroidism, rheumatoid arthritis, systemic lupus erythematosus, Sjogren’s syndrome, ankylosing spondylitis, chronic pulmonary disease, renal disease, and liver disease. An individual was defined if they received a diagnosed within 1 year before the index date for at least 2 outpatient visits or one admission.

First, a 1:4 age and sex matching was used to ensure an identical index date for any given PAOD participant with their non-PAOD counterparts. Subsequently, propensity score matching (PSM) was conducted by age, sex, and all comorbidities. The propensity score was calculated using a binary logistic regression. The binary variable was a PAOD diagnosis. PSM ensured the balanced heterogeneity of the two groups. For the subgroup analysis, the samples of the study group were divided into different subgroups according to age (20–40, 40–60, and those older than 60 years old), sex, and comorbidities (Supplementary Table S1).

### 2.4. Sensitivity Analysis

In order to verify the stability of the results, we conducted a sensitivity analysis with hypoglycemic drugs (ATC codes: A10A, A10BA, A10BB, A10BF, A10BG02, A10BG03, A10BH, A10BX02, and A10BX03) and statins (ATC code: C10AA) that were included in this study variable. Moreover, chronic limb threatening ischaemia (CLTI, ICD-9-CM = 707.10–707.15, 707.19, 730.05, 730.06, 730.07, 730.15, 730.16, 730.17, 785.4), and end-stage renal disease (ESRD, ICD-9-CM = 585 with use of erythropoietin, ATC codes: B03XA01, B03XA02, and B03XA03) were also added to control the interference of disease severity.

### 2.5. Statistical Analysis

The characteristics of the PAOD and the non-PAOD groups were compared using the absolute standardized difference (ASD). An ASD < 0.1 indicates similarity in the characteristics of both groups. The incidence density of cellulitis in the PAOD and non-PAOD groups was calculated by dividing the number of events by 1000 person-years at risk. The Kaplan–Meier curves were used to calculate the cumulative incidence of PAOD development between the study group and the control group. The relative risk (RR) of cellulitis and the confidence intervals (CI) were calculated using Poisson regression. The hazard ratio (HR) of cellulitis between the two groups was estimated by using the multivariate Cox proportional hazard model. Whether there was a significant difference between the two groups is determined by using the log-rank test. All the statistical analyzes in this study were performed in SAS version 9.4 (SAS Institute, Cary, NC, USA). Statistical significance was set at a *p*-value smaller than 0.05.

## 3. Results

### 3.1. Characteristics of the Participants

**Figure 1:**
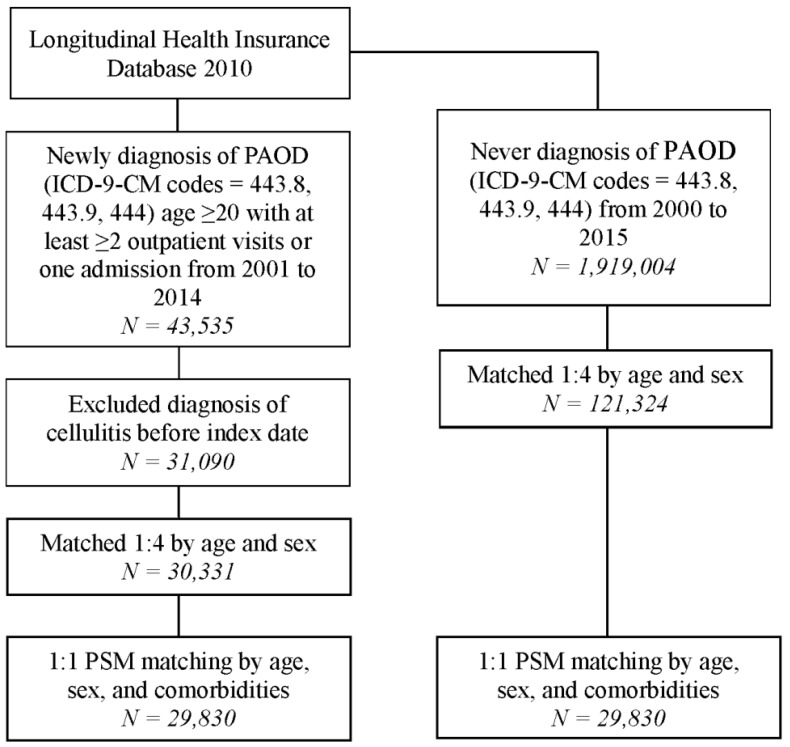
Flowchart of patient selection.

After selection, a total of 29,830 patients were included in the PAOD group (study group), while another 29,830 patients were enrolled in the non-PAOD group (control group). The study framework is shown in Figure 1. The baseline characteristics and comorbidities between the PAOD and the non-PAOD groups are shown in Table 1. The mean age of patients with and without PAOD was 63.70 ± 13.98 and 62.96 ± 14.48, respectively. The male/female ratio was similar in both groups (47.4%:52.6% in the PAOD group; 47.5%:52.5% in the non-PAOD group). There was no difference in demographic data and comorbidities between the study or control groups after the propensity score-matching process. The mean track time was 5.45 ± 4.00 and 6.07 ± 3.85 years in the PAOD and non-PAOD groups, while the time to event in the PAOD and non-PAOD groups was 3.46 ± 3.21 and 4.39 ± 3.25 years.

### 3.2. Risk of Cellulitis between PAOD and Non-PAOD Groups

**Figure 2:**
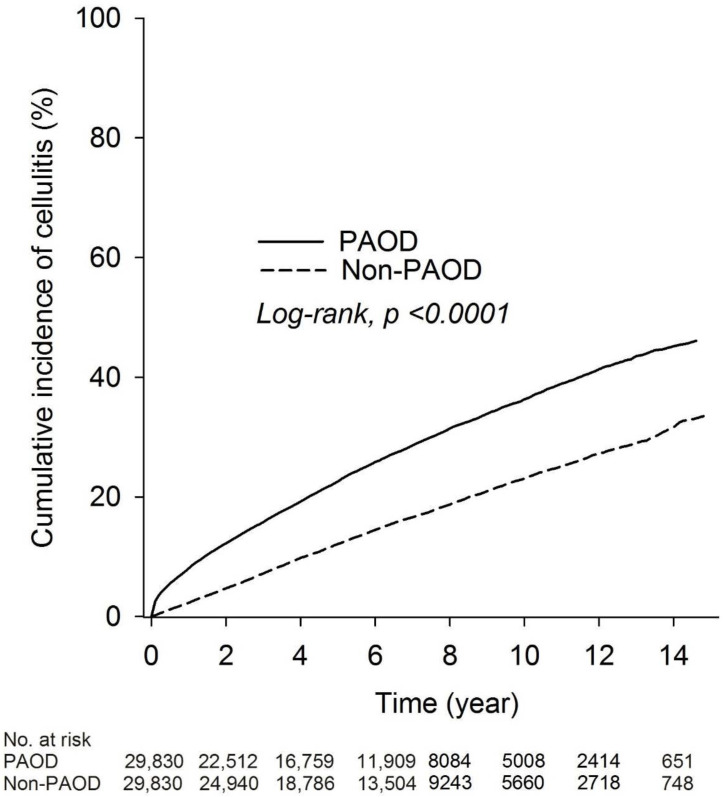
Kaplan–Meier curves of the cumulative proportions of cellulitis in PAOD patients.

The incidence densities of cellulitis were 26.05 (95% CI = 25.31–26.80) and 49.10 (95% CI = 48.04–50.19) patients per 1000 person years in the non-PAOD and PAOD groups, respectively. The RR of cellulitis was 1.89-fold (95% CI = 1.82–1.95) higher in the PAOD group compared to the non-PAOD group (Table 2). The cumulative incidence of cellulitis in both groups revealed that cellulitis was higher in the PAOD group compared to the non-PAOD group (log-rank test, *p* < 0001; Figure 2).

### 3.3. Comparison of the Risk of Cellulitis between PAOD and Non-PAOD Groups

After the statistical adjustment of age, sex, and associated comorbidities, the risk of cellulitis in the PAOD group was 1.94-fold (95% CI = 1.87–2.01) higher compared to the non-PAOD group. Compared to the subgroup aged 20–40 years old, the risk of cellulitis increased markedly with age. Compared to the female population, the risk of cellulitis was higher in the male population. The analyses of comorbidities, hypertension, diabetes, rheumatoid arthritis, SLE, ankylosing spondylitis, chronic pulmonary disease, renal disease, and liver disease as risk factors for cellulitis are shown in Table 3. In the sensitivity analysis, there were no differences between the study and control groups after the propensity score-matching process (Supplementary Table S2). After adjusting demographic data, comorbidities, and medications, the risk of cellulitis in the PAOD group was 1.85-fold (95% CI = 1.78–1.92) higher than the non-PAOD group (Supplementary Table S3).

### 3.4. Subgroup Analysis of Cellulitis Risk in the PAOD Group Relative to the Non-PAOD Group after Propensity Score Matching

In a subgroup analysis, the HR of cellulitis is higher in the PAOD group than in the non-PAOD group in all age subgroups. Compared to patients without PAOD, patients in the PAOD group aged 20 to 40 years (HR = 1.75, 95% CI = 1.48–2.08), aged 40 to 60 years (HR = 1.79; 95% CI = 1.70–1.90), and aged ≥60 years (HR = 2.12; 95% CI = 2.02–2.23) had a higher risk of cellulitis. In addition, in the PAOD group, the risk of cellulitis was higher in both male (HR = 2.12; 95% CI = 2.02–2.24) and female (HR = 1.84; 95% CI = 1.75–1.94) populations. Patients with PAOD were shown to have a higher risk of cellulitis compared to patients without PAOD, whether the following diseases were present or not: hypertension, hyperlipidemia, diabetes, hyperthyroidism, hypothyroidism, rheumatoid arthritis, Sjogren’s syndrome, chronic pulmonary disease, renal disease, and liver disease. Furthermore, the risk of cellulitis was significantly higher in the PAOD group compared to the non-PAOD group for patients without the comorbidity of ankylosing spondylitis. The details of the subgroup analyses are shown in Table 4.

## 4. Discussion

Patients with or without a new diagnosis of PAOD were enrolled to analyze their association with cellulitis. In this study, we found that the incidence of cellulitis increased in patients with PAOD compared to those without PAOD. PAOD is associated with atherosclerosis, which results in the subintimal accumulation of lipid and fibrous materials and causes the vessel lumen to narrow. PAOD is often asymptomatic, and symptoms of PAOD such as claudication, rest pain, ulceration, and gangrene are mainly based on the extent of vascular narrowing and the locations of affected vessels. In patients with PAOD, the adhesion and recruitment of leukocyte and platelet cells, increased activity of platelet activation and thrombosis formation, and dysregulation of vessel tone may cause endothelial dysfunction [23,24]. The selective barrier function of regulating the transport of fluid and intercellular components, vascular reactivity via NO synthase to control blood flow, and proinflammatory activation are considered vital functions of endothelial cells [24,25,26,27,28]. Dysfunction of endothelial cells affects the regulation of coagulation, platelet, and complement activation, as well as leukocyte recruitment in the microvasculature [29]. The progressive reduction of blood flow also gives rise to free radical production and lower oxygen supplementation [30]. Oxygen is important for wound healing, cellular function maintenance, and prophylaxis against infection [31]. Inadequate perfusion of skin tissue below the level adequate to maintain skin structure may change the physiological integrity of skin tissue and, finally, lead to poor wound healing, an ischemic ulcer, or gangrene. The alteration of the immune defenses and destruction of skin integrity result in an increasing risk of developing infection.

Several studies have documented the association between PAOD and infectious diseases. Wang et al. revealed that patients with PAOD were at an increased risk of future sepsis events [32]. The other retrospective cohort study in 2022 showed a similar finding; it reported patients with PAOD were prone to suffer from impaired immune responses due to endothelial damage and dysfunction. Those patients also had a higher risk of sepsis after infection [33]. Kim et al. found that when admitted with an infection of SARS-CoV-2, patients with PAOD have over 40% mortality [34]. Moreover, an observational cohort study in the United States during the period of 2011–2013, which enrolled patients aged 71 to 90 years, reported that the contribution of PAOD to infection risk was similar to that of coronary heart disease and stroke, and PAOD was firmly associated with infection-related hospitalization in older individuals [35]. By contrast, we found that patients with PAOD were associated with a higher HR of cellulitis in all age subgroups.

A retrospective study in Sweden from 2005 to 2010 indicated the rate of infection was significantly associated with the degree of lower extremity ischemia [36]. This study has clinical implications. Patients with PAOD have a high risk of cellulitis. The length of time from the index date to the diagnosis of cellulitis is shorter in the PAOD group than in the non-PAOD group (3.46 ± 3.21 vs 4.39 ± 3.25 years). Skin necrosis, ulcers, gangrene, or chronic limb-threatening ischemia may be represented as the progression of PAOD and the susceptibility to infection. In addition, coexistence with complex wounds and superimposed infections also increases the difficulty of treatments. On the other hand, severe PAOD may develop chronic limb-threatening ischemia (CLTI), which has a high rate of mortality and limb loss. The risk of limb loss in patients without treatment is estimated to be about 20% by 1 year, and the mortality rate is typically over 50% by 5 years [37,38]. It is predictable that PAOD will become increasingly universal in the future with aging of the global population. Despite the fact that it is extremely common, the early stage of PAOD is often asymptomatic, and the majority of PAOD patients are undiagnosed and undertreated [39]. Identifying the risk factors and paying attention to the early detection of PAOD are imperative. Lifestyle modifications, such as smoking cessation, weight reduction, and treatment of other metabolic or cardiovascular diseases, can reduce rates of adverse events and complications [40,41]. To prevent further infections and failures in wound healing, angioplasty, bypass surgery, and hyperbaric oxygen therapy should also be considered [42,43,44].

There are several limitations to this study. First, the physiological and laboratory data of the studied patients were not contained in the database. We cannot evaluate the severity and process of infectious diseases in the absence of C-reactive protein (CRP), erythrocyte sedimentation rate (ESR) level, the results of blood or pus culture, or the ankle-brachial index (ABI), which is a non-invasive assessment tool used to measure the severity of atherosclerotic burden. CRP and ESR are two frequently ordered laboratory tests that may help physicians diagnose and access disease states. However, in some cases, CRP and ESR were not recommended as tools used to alter medical decision-making and the course of treatment in a patients with an indeterminate clinical suspicion of disease because of low sensitivity or specificity [45]. On the other hand, ABI is considered a useful, simple, and inexpensive tool for the diagnosis and assessment of the severity of PAOD; however, it has a wide range of sensitivity, especially in elderly individuals and patients with diabetes [46,47]. Second, the laboratory markers of disease severity of risk factors for cellulitis, such as the glycated hemoglobin (HBA1c) level in diabetes and renal function in chronic kidney disease, cannot be collected from the claims database. The site of ulcer or gangrene and the region of cellulitis were also not fully obtained. It was hard to determine the time sequence and analyze the existing association between soft tissue lesions such as venous ulcers or lymphedema and cellulitis. In addition, we did not know the mechanism of cellulitis related to open wounds, the distribution of cellulitis at each stage of PAOD, or the proportion of undiagnosed PAOD patients in the control group in this study, which cannot answer whether patients with each stage of PAOD were associated a higher risk of subsequent cellulitis compared to patients without PAOD. Third, the database did not provide the timing of the intervention, such as debridement, and the differences in therapeutic strategies between PAOD and non-PAOD groups were not compared. In order to conquer these limitations, we used a propensity score by matching age, sex, and comorbidities to reduce the heterogeneity between the two groups. Fourth, this is an observational and retrospectively designed study, and the accuracy and precision of the results may be restricted. The exact mechanism linking the correlations between PAOD and cellulitis is required to be clarified by further investigation.

## 5. Conclusions

In conclusion, the results of this study revealed that patients with PAOD were associated with a higher risk of subsequent cellulitis compared to patients without PAOD. PAOD had a significant risk of cellulitis among patients who were ≥60 years old, male, had diabetes, had ankylosing spondylitis, and were in the renal disease subgroup.

## Figures and Tables

**Table 1:** Demographic characteristics of PAOD and non-PAOD.

	Before PSM Matching					After PSM Matching				
	Non-PAOD *(N* = 121,324)		PAOD (*N* = 30,331)			PAOD (*N* = 29,830)		Non-PAOD (*N* = 29,830)		
	*n*	%	*n*	%	ASD	*n*	%	*n*	%	ASD
Age					<0.001					<0.001
20–40	8312	6.9	2078	6.9		1944	6.5	2050	6.9	
40–60	54,332	44.8	13,583	44.8		13,146	44.1	13,255	44.4	
≥60	58,680	48.4	14,670	48.4		14,740	49.4	14,525	48.7	
Mean ± SD	62.95 ± 14.48		62.95 ± 14.48		<0.001	63.70 ± 13.98		62.96 ± 14.48		0.063
Sex					<0.001					0.003
Female	63,468	52.3	15,867	52.3		15,700	52.6	15,653	52.5	
Male	57,856	47.7	14,464	47.7		14,130	47.4	14,177	47.5	
Hypertension	36,460	30.1	14,700	48.5	0.384	14,394	48.3	14,216	47.7	0.012
Hyperlipidemia	13,633	11.2	5863	19.3	0.226	5873	19.7	5742	19.2	0.011
Diabetes	14,823	12.2	8382	27.6	0.393	7908	26.5	7904	26.5	<0.001
Hyperthyroidism	720	0.6	238	0.8	0.023	247	0.8	234	0.8	0.005
Hypothyroidism	511	0.4	172	0.6	0.021	170	0.6	165	0.6	0.002
Rheumatoid arthritis	668	0.6	356	1.2	0.067	350	1.2	344	1.2	0.002
Systemic lupus erythematosus	88	0.1	75	0.2	0.044	56	0.2	60	0.2	0.003
Sjogren’s syndrome	465	0.4	212	0.7	0.043	222	0.7	206	0.7	0.006
Ankylosing spondylitis	133	0.1	74	0.2	0.032	62	0.2	73	0.2	0.008
Chronic pulmonary disease	9898	8.2	3823	12.6	0.146	3863	13.0	3746	12.6	0.012
Renal disease	2625	2.2	2576	8.5	0.285	1973	6.6	2080	7.0	0.014
Liver disease	4946	4.1	2012	6.6	0.114	2042	6.8	1972	6.6	0.009

ASD: absolute standardized differences; PAOD: peripheral arterial occlusion disease.

**Table 2:** Poisson regression of the relative risk of PAOD and non-PAOD.

	Non-PAOD	PAOD
*N*	29,830	29,830
Person-years	180,947	162,677
No. of cellulitis	4713	7988
ID (95% CI)	26.05 (25.31–26.80)	49.10 (48.04–50.19)
Relative risk (95% CI)	Reference	1.89 (1.82–1.95)

ID: incidence density (per 1000 person-years); CI: confidence interval; PAOD: peripheral arterial occlusion disease.

**Table 3:** Cox proportional hazard model analysis for the risk of cellulitis.

	Univariate		Multivariate †	
	HR (95% CI)	*p* Value	HR (95% CI)	*p* Value
Group				
Non-PAOD	Reference		Reference	
PAOD	1.87 (1.81–1.94)	<0.001	1.94 (1.87–2.01)	<0.001
Age				
20–40	Reference		Reference	
40–60	1.48 (1.36–1.61)	<0.001	1.30 (1.19–1.41)	<0.001
≥60	2.31 (2.12–2.51)	<0.001	1.84 (1.69–2.01)	<0.001
Sex				
Female	Reference		Reference	
Male	1.16 (1.12–1.21)	<0.001	1.14 (1.10–1.18)	<0.001
Hypertension	1.42 (1.37–1.47)	<0.001	1.13 (1.09–1.17)	<0.001
Hyperlipidemia	1.12 (1.07–1.17)	<0.001	0.91 (0.87–0.95)	<0.001
Diabetes	1.74 (1.68–1.81)	<0.001	1.62 (1.56–1.69)	<0.001
Hyperthyroidism	0.90 (0.74–1.11)	0.324	0.97 (0.79–1.18)	0.734
Hypothyroidism	0.99 (0.77–1.28)	0.949	0.99 (0.77–1.27)	0.923
Rheumatoid arthritis	1.43 (1.24–1.65)	<0.001	1.40 (1.21–1.62)	<0.001
Systemic lupus erythematosus	1.57 (1.13–2.18)	0.007	1.78 (1.28–2.47)	<0.001
Sjogren’s syndrome	1.14 (0.92–1.40)	0.227	1.05 (0.86–1.30)	0.621
Ankylosing spondylitis	1.68 (1.24–2.26)	<0.001	1.70 (1.26–2.30)	<0.001
Chronic pulmonary disease	1.44 (1.37–1.51)	<0.001	1.26 (1.20–1.32)	<0.001
Renal disease	1.87 (1.75–1.99)	<0.001	1.59 (1.50–1.70)	<0.001
Liver disease	1.18 (1.10–1.26)	<0.001	1.10 (1.03–1.18)	0.003

HR: hazard ratio; CI: confidence interval; PAOD: peripheral arterial occlusion disease; † adjusted for age, sex, hypertension, hyperlipidemia, diabetes, hyperthyroidism, hypothyroidism, rheumatoid arthritis, systemic lupus erythematosus, Sjogren’s syndrome, ankylosing spondylitis, chronic pulmonary disease, renal disease, and liver disease.

**Table 4:** Subgroup analysis of the risk of cellulitis.

	Non-PAOD		PAOD			
	*N*	No. of Cellulitis Cases	*N*	No. of Cellulitis Cases	HR (95% CI)	*p* Value
Age ^1^						
20–40	1944	210	2050	379	1.75 (1.48–2.08)	<0.001
40–60	13,146	1935	13,255	3172	1.79 (1.70–1.90)	<0.001
≥60	14,740	2568	14,525	4437	2.12 (2.02–2.23)	<0.001
*p* for interaction < 0.001						
Sex ^1^						
Female	15,700	2499	15,653	3997	1.84 (1.75–1.94)	<0.001
Male	14,130	2214	14,177	3991	2.12 (2.02–2.24)	<0.001
*p* for interaction < 0.001						
Hypertension ^1^						
No	15,436	2235	15,614	3791	1.91 (1.82–2.02)	<0.001
Yes	14,394	2478	14,216	4197	2.03 (1.93–2.13)	<0.001
*p* for interaction = 0.0640						
Hyperlipidemia ^1^						
No	23,957	3804	24,088	6428	1.97 (1.89–2.05)	<0.001
Yes	5873	909	5742	1560	2.01 (1.85–2.18)	<0.001
*p* for interaction = 0.5347						
Diabetes ^1^						
No	21,922	3308	21,926	5187	1.75 (1.68–1.83)	<0.001
Yes	7908	1405	7904	2801	2.49 (2.34–2.66)	<0.001
*p* for interaction < 0.001						
Hyperthyroidism ^2^						
No	29,583	4673	29,596	7935	1.98 (1.91–2.05)	<0.001
Yes	247	40	234	53	1.69 (1.11–2.56)	0.015
*p* for interaction = 0.4887						
Hypothyroidism ^3^						
No	29,660	4689	29,665	7951	1.97 (1.90–2.05)	<0.001
Yes	170	24	165	37	2.04 (1.19–3.49)	0.010
*p* for interaction = 0.8925						
Rheumatoid arthritis ^1^						
No	29,480	4640	29,486	7869	1.97 (1.90–2.04)	<0.001
Yes	350	73	344	119	2.11 (1.57–2.84)	<0.001
*p* for interaction = 0.4890						
Systemic lupus erythematosus ^4^						
No	29,774	4698	29,770	7967	1.98 (1.91–2.05)	<0.001
Yes	56	15	60	21	1.56 (0.68–3.56)	0.292
*p* for interaction = 0.7374						
Sjogren’s syndrome ^5^						
No	29,608	4672	29,624	7940	1.98 (1.91–2.05)	<0.001
Yes	222	41	206	48	1.61 (1.04–2.50)	0.034
*p* for interaction = 0.2626						
Ankylosing spondylitis ^6^						
No	29,768	4691	29,757	7967	1.98 (1.91–2.05)	<0.001
Yes	62	22	73	21	1.15 (0.60–2.20)	0.680
*p* for interaction = 0.0161						
Chronic pulmonary disease ^1^						
No	25,967	3973	26,084	6787	1.96 (1.88–2.04)	<0.001
Yes	3863	740	3746	1201	2.05 (1.87–2.25)	<0.001
*p* for interaction = 0.2229						
Renal disease ^1^						
No	27,857	4362	27,750	7278	1.92 (1.85–2.00)	<0.001
Yes	1973	351	2080	710	2.65 (2.33–3.02)	<0.001
*p* for interaction <0.001						
Liver disease ^1^						
No	27,788	4339	27,858	7387	1.98 (1.90–2.05)	<0.001
Yes	2042	374	1972	601	1.94 (1.71–2.21)	<0.001
*p* for interaction = 0.8008						

^1^ Adjusted for age, sex, and comorbidities. ^2^ Adjusted for age, sex, and comorbidities, excluding ankylosing spondylitis. ^3^ Adjusted for age, sex, and comorbidities, excluding systemic lupus erythematosus, Sjogren’s syndrome, and ankylosing spondylitis. ^4^ Adjusted for age, sex, and comorbidities, excluding hypothyroidism, and ankylosing spondylitis. ^5^ Adjusted for age, sex, and comorbidities, excluding hypothyroidism. ^6^ Adjusted for age, sex, and comorbidities, excluding hyperthyroidism, and systemic lupus erythematosus.

## Data Availability

The data used in this study were obtained from the National Health Insurance database and are available from the authors with permission of the National Health Insurance Administration of Taiwan, subject to certain restrictions.

## Supplementary Materials

The following supporting information can be downloaded at: https://www.mdpi.com/article/10.3390/medicina59050933/s1, Table S1: Disease code of comorbidities; Table S2: Sensitivity analysis of demographic characteristics of PAOD and Non-PAOD; Table S3: Sensitivity analysis of Cox proportional hazard model analysis for risk of cellulitis.
