# Supplementary material for: Evaluation of the Risk Factors for Cellulitis among Patients with Peripheral Artery Disease
Source: Medicina (Kaunas). 2023 May 12;59(5):933. doi: 10.3390/medicina59050933 (PMC10224094; doi:10.3390/medicina59050933)
Supplement: Supplementary file 1 [file medicina-59-00933-s001.zip › medicina-2359479-supplementary.pdf]

Supplementary Table S1. Disease code of comorbidities.

Disease	ICD-9-CM
Hypertension	401–405
Hyperlipidemia	272.0–272.4
Diabetes	250
Hyperthyroidism	242
Hypothyroidism	243, 244
Rheumatoid arthritis	714.0
Systemic lupus erythematosus	710.0
Sjogren’s syndrome	710.2
Ankylosing spondylitis	720.0
Chronic pulmonary disease	490–496, 500–505, 506.4
Renal disease	582, 583.0–583.7, 585, 586, 588
Liver disease	456.0–456.21, 571.2, 571.4, 571.5, 571.6, 572.2–572.8

Supplementary Table S2. Sensitivity analysis of demographic characteristics of PAOD and Non-PAOD.

	Before PSM matching					After PSM matching				
	Non-PAOD (N = 121324)		PAOD (N =30331)			PAOD (N = 29007)		Non-PAOD (N = 29007)		
	n	%	n	%	ASD	n	%	n	%	ASD
Age					<0.001					<0.001
20-40	8312	6.9	2078	6.9		1915	6.6	2023	7.0	
40-60	54332	44.8	13583	44.8		12964	44.7	13001	44.8	
≥60	58680	48.4	14670	48.4		14128	48.7	13983	48.2	
Mean ± SD	62.95 ± 14.48		62.95 ± 14.48		<0.001	63.75 ± 14.03		62.84 ± 14.55		0.063
Sex					<0.001					0.008
Female	63468	52.3	15867	52.3		15380	53.0	15269	52.6	
Male	57856	47.7	14464	47.7		13627	47.0	13738	47.4	
Hypertension	36460	30.1	14700	48.5	0.384	13849	47.7	13702	47.2	0.010
Hyperlipidemia	13633	11.2	5863	19.3	0.226	5622	19.4	5562	19.2	0.005
Diabetes	14823	12.2	8382	27.6	0.393	7387	25.5	7384	25.5	<0.001
Hyperthyroidism	720	0.6	238	0.8	0.023	227	0.8	229	0.8	0.001
Hypothyroidism	511	0.4	172	0.6	0.021	157	0.5	159	0.5	0.001
Rheumatoid Arthritis	668	0.6	356	1.2	0.067	351	1.2	335	1.2	0.005
Systemic lupus erythematosus	88	0.1	75	0.2	0.044	57	0.2	59	0.2	0.002
Sjogren's syndrome	465	0.4	212	0.7	0.043	209	0.7	203	0.7	0.002
Ankylosing spondylitis	133	0.1	74	0.2	0.032	62	0.2	69	0.2	0.005
Chronic pulmonary disease	9898	8.2	3823	12.6	0.146	3634	12.5	3620	12.5	0.001
Liver disease	4946	4.1	2012	6.6	0.114	2001	6.9	1912	6.6	0.012
CLTI	66	0.1	701	2.3	0.210	66	0.2	67	0.2	0.001
Hypoglycemic drugs	14197	11.7	8121	26.8	0.390	7195	24.8	7153	24.7	0.003
Statin	11259	9.3	5206	17.2	0.234	4896	16.9	4824	16.6	0.007
ESRD	446	0.4	1217	4.0	0.251	446	1.5	489	1.7	0.012

ASD: Absolute standardized difference; PAOD: Peripheral Arterial Occlusion Disease; CLTI: Chronic limb Threatening ischaemia; ESRD: End-Stage Renal Disease.

Supplementary Table S3. Sensitivity analysis of Cox proportional hazard model analysis for risk of cellulitis.

	Univariate		Multivariate†	
	HR (95% C.I.)	p value	HR (95% C.I.)	p value
Group				
Non-PAOD	Reference		Reference	
PAOD	1.79 (1.73-1.86)	<.0001	1.85 (1.78-1.92)	<.0001
Age				
20-40	Reference		Reference	
40-60	1.47 (1.35-1.61)	<.0001	1.29 (1.18-1.41)	<.0001
≥60	2.30 (2.11-2.51)	<.0001	1.85 (1.69-2.02)	<.0001
Sex				
Female	Reference		Reference	
Male	1.16 (1.12-1.21)	<.0001	1.14 (1.10-1.18)	<.0001
Hypertension	1.40 (1.35-1.45)	<.0001	1.12 (1.08-1.16)	<.0001
Hyperlipidemia	1.12 (1.07-1.18)	<.0001	0.93 (0.88-0.99)	0.013
Diabetes	1.68 (1.62-1.75)	<.0001	1.14 (1.04-1.24)	0.004
Hyperthyroidism	1.04 (0.85-1.27)	0.691	1.13 (0.93-1.38)	0.227
Hypothyroidism	1.03 (0.79-1.33)	0.838	1.02 (0.79-1.32)	0.891
Rheumatoid Arthritis	1.42 (1.23-1.64)	<.0001	1.38 (1.19-1.60)	<.0001
Systemic lupus erythematosus	1.42 (1.01-1.99)	0.041	1.63 (1.17-2.29)	0.005
Sjogren's syndrome	1.11 (0.90-1.38)	0.341	1.05 (0.84-1.30)	0.681
Ankylosing spondylitis	1.70 (1.25-2.32)	<0.001	1.69 (1.24-2.29)	<0.001
Chronic pulmonary disease	1.46 (1.39-1.53)	<.0001	1.29 (1.22-1.35)	<.0001
Liver disease	1.17 (1.09-1.25)	<.0001	1.11 (1.04-1.19)	0.003
CLTI	4.30 (3.33-5.55)	<.0001	3.79 (2.93-4.90)	<.0001
Hypoglycemic drugs	1.72 (1.65-1.79)	<.0001	1.43 (1.31-1.56)	<.0001
Statin	1.20 (1.14-1.25)	<0.001	1.00 (0.94-1.06)	0.938
ESRD	2.12 (1.87-2.41)	<.0001	2.01 (1.77-2.28)	<.0001

HR: Hazard Ratio; CI: Confidence Interval; PAOD: Peripheral Arterial Occlusion Disease; CLTI: Chronic limb Threatening ischaemia; ESRD: End-Stage Renal Disease; †Adjusted for age, sex, hypertension, hyperlipidemia, diabetes, hyperthyroidism, hypothyroidism, rheumatoid arthritis, systemic lupus erythematosus, Sjogren's syndrome, ankylosing spondylitis, chronic pulmonary disease, liver disease, chronic limb threatening ischaemia, hypoglycemic drugs, statin, and end-stage renal disease.
